# Nuclear DNA Replication in Trypanosomatids: There Are No Easy Methods for Solving Difficult Problems

**DOI:** 10.1016/j.pt.2017.08.002

**Published:** 2017-11

**Authors:** Marcelo S. da Silva, Raphael S. Pavani, Jeziel D. Damasceno, Catarina A. Marques, Richard McCulloch, Luiz Ricardo Orsini Tosi, Maria Carolina Elias

**Affiliations:** 1 Laboratório Especial de Ciclo Celular (LECC), Center of Toxins, Immune Response and Cell Signaling (CeTICS), Butantan Institute, São Paulo, SP, Brazil; 2 Departamento de Biologia Celular e Molecular e Bioagentes Patogênicos, Faculdade de Medicina de Ribeirão Preto, Universidade de São Paulo (USP), Ribeirão Preto, SP, Brazil; 3 Division of Biological Chemistry and Drug Discovery, School of Life Sciences, Dow Street, University of Dundee, Dundee, UK; 4 The Wellcome Centre for Molecular Parasitology, Institute of Infection, Immunity and Inflammation, University of Glasgow, Glasgow, UK

**Keywords:** DNA replication, origin recognition complex, replication fork, replication origins, replication stress, trypanosomatid emergence

## Abstract

In trypanosomatids, etiological agents of devastating diseases, replication is robust and finely controlled to maintain genome stability and function in stressful environments. However, these parasites encode several replication protein components and complexes that show potentially variant composition compared with model eukaryotes. This review focuses on the advances made in recent years regarding the differences and peculiarities of the replication machinery in trypanosomatids, including how such divergence might affect DNA replication dynamics and the replication stress response. Comparing the DNA replication machinery and processes of parasites and their hosts may provide a foundation for the identification of targets that can be used in the development of chemotherapies to assist in the eradication of diseases caused by these pathogens.

## Trends

In trypanosomatids, DNA replication is tightly controlled by protein complexes that diverge from those of model eukaryotes.

There is no consensus for the number of replication origins used by trypanosomatids; how their replication dynamics compares with that of model organisms is the subject of debate.

The DNA replication rate in trypanosomatids is similar to, but slightly higher than, that of model eukaryotes, which may be related to chromatin structure and function.

Recent data suggest that the origin recognition complex in trypanosomatids closely resembles the multisubunit eukaryotic model.

The absence of fundamental replication-associated proteins in trypanosomatids suggests that new signaling pathways may be present in these parasites to direct DNA replication and the replicative stress response.

## Trypanosomatids, a Group of Eukaryotes with Peculiar Features

**Figure 1:**
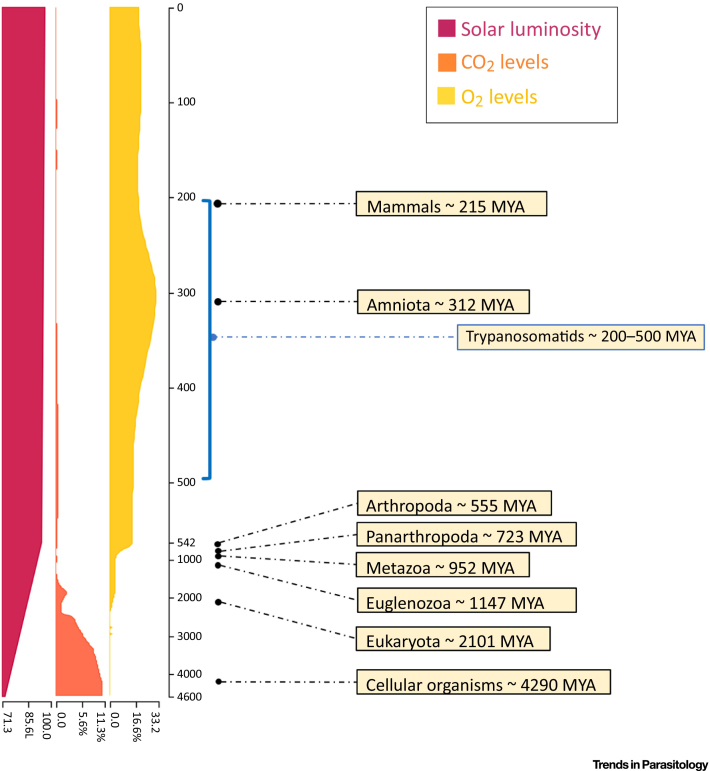
Evolutionary Timeline of Eukaryotes. Trypanosomatids diverged around 200–500 million years ago (MYA), probably due to the emergence of new niches precipitated by the appearance of the metazoans, which allowed coevolution of trypanosomatids with both vertebrate and arthropod hosts. Adapted and modified with permission from www.timetree.org.

Trypanosomatids are a group of parasitic single-celled eukaryotes within the order Kinetoplastea. Amongst the trypanosomatids are human pathogens of paramount medical importance, such as *Leishmania* spp. (etiological agent of distinct forms of leishmaniasis), *Trypanosoma cruzi* (etiological agent of Chagas’ disease), and *Trypanosoma brucei* (etiological agent of African sleeping sickness). Altogether, these parasites are responsible for more than 50 000 deaths annually [1]. Trypanosomatids present a heteroxenous life cycle (i.e., they require more than one host to complete their life cycle), varying between replicative (usually noninfective) and nonreplicative (infective) forms, which makes one wonder if genome replication and infection could be mutually exclusive events. They diverged from other eukaryotes around 200–500 million years ago (MYA) 2, 3, 4, which comprises the period between the emergence of arthropods and mammals (Figure 1). This timing suggests that trypanosomatids diverged as a result of new niches provided by the metazoans, which allowed trypanosomatids to coevolve with them and led to the emergence of parasitic and symbiotic relationships 3, 4, 5. Associated with this evolution, trypanosomatids present several unique characteristics amongst eukaryotes, including the near universal use of multigenic RNA polymerase (Pol) II transcription and, in *T. brucei*, adaptation of RNA Pol I to transcribe genes encoding surface proteins. These peculiar features might be explained by the fact that these organisms belong to the phylum Euglenozoa, maintaining the ‘primitive’ characteristics of this higher order taxon, or by genome streamlining, since the transition from a free-living to a parasitic lifestyle resulted in the loss of many protein-coding genes in these organisms 6, 7. Whether the unusual composition and structure of at least some of the proteins involved in DNA replication in trypanosomatids, as well as the unique dynamics of the reaction, reflect ancient peculiarities or adaptive events remains unclear.

In general, the dynamics and mechanics of DNA replication are conserved among living organisms and fine-tuned to cellular growth. In eukaryotes, at the end of mitosis and during the G1 phase, protein complexes are assembled onto sites in the DNA genome, establishing these locations as potential regions for the onset of new DNA synthesis (these regions are called replication origins). When the S phase is reached, some of these established origins will be activated, at which time DNA replication proceeds by the recruitment of further protein complexes. Understanding the processes involved in trypanosomatid DNA replication could not merely provide an evolutionary perspective on this central cellular reaction but may help to explain the striking ability of these parasites to gain, lose, or rearrange DNA, allowing for a better adaptation to the environment 8, 9. Knowledge about genome maintenance pathways, and in particular the process of nuclear DNA replication, in any trypanosomatids, is limited compared to that of model eukaryotes. However, several studies have emerged recently that have disclosed many unusual features of the replication machinery, the control of re-replication and the replication stress responses, elevating trypanosomatid parasites to the forefront of understanding of nuclear DNA replication and transmission amongst protozoans, which provide much of the diversity of the eukaryotic domain of life. In this review, we focus on the main advances in recent years regarding the identification of replication origins, the structure of the protein complexes involved in nuclear DNA replication, and mechanisms to avoid re-replication and cope with replicative stress in the main pathogenic trypanosomatids. Moreover, by comparing these processes with what is known in model eukaryotes, we hope to leave clues in a trail of discovery that will ask if important components or controls of nuclear DNA replication could emerge as a target for the development of new antitrypanosomatid therapies, helping to eradicate the diseases caused by these parasites.

## DNA Replication Origins

DNA replication origins are strictly defined as sites in the DNA genome that are bound by specialized initiator proteins in order that the wider replication machinery can be loaded and the onset of genomic DNA synthesis can begin. In general, bacteria typically have a single replication origin per genome, archaea can have a single origin or can use a few origins, whereas each model eukaryote genome is replicated from hundreds or thousands of origins. Currently, there is little clear consensus for the number of replication origins used by trypanosomatids, meaning that how their replication dynamics compare with prokaryotes and eukaryotes is the subject of debate.

The first study in trypanosomatids to identify and count the number of DNA replication origins was a genome-wide analysis performed in *T. brucei* using a technique called MFAseq (Box 1) [10]. This assay showed that *T. brucei*, like all previously characterized eukaryotes, presents multiple origins per chromosome, with peak amplitude variation, as obtained by MFAseq, suggesting differing timing or frequency of activation. However, *T. brucei* origins are more widely spaced than in other eukaryotes: one origin for each 260 kb, compared with budding yeast, where there is one per every 46 kb, and mammalian cells, where there is one every 25 to 130 kb [10]. Nonetheless, correlating MFAseq peak location and the binding sites of a replication-initiating factor (see below) showed that *T. brucei* licenses more origins than are activated during the S phase. Though origins and initiator-binding sites all localize to the ends of the multigene transcription units, no consensus sequence for origins was found [10].

**Figure I:**
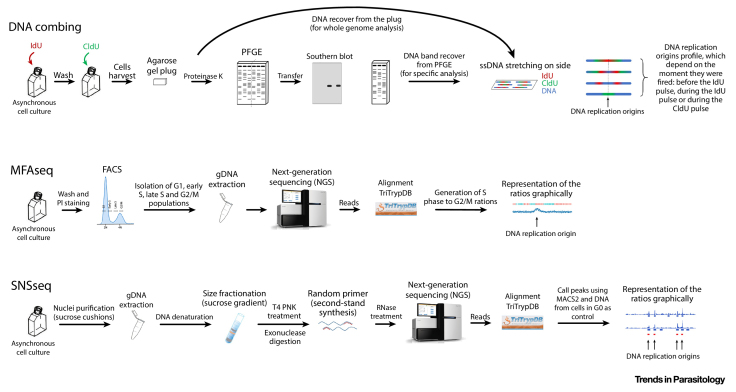
The Main Steps of the Techniques Used to Monitor DNA Replication in Trypanosomatids. DNA combing, MFAseq, and SNSseq. IdU, 5-iodo-2′-deoxyuridine; CldU, 5-chloro-2′-deoxyuridine; PFGE, pulsed-field gel electrophoresis; ssDNA, single-stranded DNA; FACS, fluorescence-activated cell sorting; gDNA, genomic DNA; NGS, next-generation sequencing; T4 PNK, polynucleotide kinase from T4 bacteriophage. Of note, these key steps were described in previous studies 12, 13, 14, 21, 24.

Box 1 **Techniques Used to Monitor DNA Replication Origins in Trypanosomatids**

**DNA Combing**

This technique is used to produce an array of uniformly stretched DNA molecules, allowing the investigation of DNA replication on single molecules. It requires two consecutive pulses of thymidine analogs (usually IdU and CldU) in an asynchronous culture of cells. Usually, the cells with IdU–CldU incorporated are trapped in agarose plugs and the DNA is isolated by treatment with proteinase K. For analysis of a specific fragment of the genome, the plug containing DNA is subjected to pulsed-field gel electrophoresis (PFGE). Part of this PFGE is submitted to Southern blotting, which, will detect a fragment of interest through hybridization with specific probes. The fragment of interest is then recovered from the other part of PGFE, treated, and stretched (combed) on slides. Alternatively, for an analysis of the whole genome, the plug containing DNA treated with proteinase K can be directly stretched (combed) on the slides. IdU, CldU, and DNA are detected by indirect immunofluorescence using specific antibodies. Different signal patterns for DNA replication origins can be observed in the slide analysis, allowing visualization of replication fork direction, including initiation and termination regions (Figure I).

**MFAseq**

‘Marker frequency analysis coupled with deep sequencing’, also termed Sort-seq in yeast and archaea, is a population-based analysis in which DNA read depth across the genome of replicating cells is compared relative to that in nonreplicating cells. This results in a landscape of the replication profile across the genome, in which ‘peaks’ represent replicating regions of the chromosome (regions where DNA replication origins are inferred to be present), while ‘valleys’ (representing the convergence of two replication forks) suggest replication termination zones. Moreover, the peak height and width can also provide information on origin usage and replication speed. Briefly, an asynchronous culture of cells is fixed and stained with a DNA dye (e.g., propidium iodide) and sorted by fluorescence-activated cell sorting (FACS) into populations of the different cell cycle stages. Sorting the S phase into two populations, early and late, is optional but allows the analysis of the replication profile at two different stages, and the mapping of early and late replicating regions of the genome. Once the populations have been isolated, genomic DNA is extracted, prepared for next-generation sequencing (NGS), and sequenced. The resulting reads are then aligned to the reference genome (which can be retrieved from TriTrypDB.org) and the frequency of reads (coverage) is assessed per base pair, which in this analysis acts as a ‘marker’. For each population, the genome is then ‘fragmented’ into bins (analysis to date used 1–2.5 kbp bins) and the median of the coverage per bin is calculated (for each sample, the median of read depth per bin is compared to the general genome-wide average of reads of that sample). Coverage is then used to compute the ratio between the replicating populations (S phase) and the nonreplicating ones (G1 or G2/M phases) per bin, which is then plotted and represented graphically across the genome (Figure I).

**SNSseq**

‘Small nascent strand purification coupled with deep sequencing’ is a highly sensitive, population-based analysis for sequencing initiation-proximal nascent DNA strands that are linked to RNA primers (Figure I) and is widely used in studies of replication in metazoans; it is capable of detecting initiation sites used by only a few cells in the population. Simplistically, nuclei are purified from an asynchronous culture of cells using sucrose cushions, and the genomic DNA is extracted. The DNA is then heat-denaturated and size-fractionated using a range of sucrose gradients. Fragments ranging from 300 to 1500 nucleotides are then purified and subjected to rounds of T4 polynucleotide kinase (PNK) phosphorylation, followed by digestion with λ-exonuclease. The treated DNA is then subjected to random-primed second-strand synthesis and next digested with RNase. The resulting DNA is then processed to generate next-generation sequencing (NGS) libraries, and sequenced. The reads obtained are then aligned to the reference genome, and peaks are called using the callpeak function of MACS2 (using processed DNA from cells collected from a cell culture in stationary phase as a control). The peak-profile can then be plotted and represented graphically across the genome (Figure I).

Alt-text: Box 1

Remarkably, when MFAseq was applied to *Leishmania major* and *Leishmania mexicana*, only a single replication initiation site per chromosome was found [11], an unprecedented observation in eukaryotes. Considering that trypanosomatids perform polycistronic transcription and there might be transcription during replication, the process of origin activation in these parasites might need to strike an equilibrium between the activation of as few origins as possible to limit replication–transcription conflicts, and the activation of sufficient origins to allow replication of the entire genome. In this scenario, it appears that different solutions have been reached by each parasite. However, two other techniques may indicate that there are more origins activated in *T. brucei* and *Leishmania* spp. than are detected by MFAseq. One of these techniques is called DNA combing (Box 1), which was used to visualize replication in a specific region of *T. brucei* chromosome 1, revealing initiation at a single non-MFAseq mapped initiator binding site, and therefore origin, after hydroxyurea treatment [12]. DNA combing was also applied to undefined DNA molecules in *Leishmania* spp. and *T. brucei*, revealing more than a single initiation site in *Leishmania* spp and less widely spaced initiation sites in *T. brucei*, but without correlation to initiator-binding sites or wider chromosome features [13]. The other approach used, called SNSseq (Box 1), revealed more than 5000 sites of replication initiation throughout the *Leishmania* chromosomes, with most sites dispersed throughout the multigene transcription units [14].

The DNA combing and SNSseq studies may suggest that there are more active origins in *T. brucei* and *Leishmania* spp. than those detected by MFAseq, which provides an indication of the frequency with which an origin is activated in a cell population 10, 11, meaning that MFAseq might detect mainly **constitutive origins** (see Glossary). Indeed, some authors have suggested that MFAseq peaks actually represent sites of pronounced concentration of many origins 14, 15. In contrast, DNA combing and SNSseq techniques may not reflect the frequency of origin activation, meaning that these techniques might also identify **flexible, possiblydormant origins**. However, it is important to stress than only MFAseq has, to date, been correlated with initiator binding, so it is also possible that unconventional, origin-independent replication initiation events are also being detected by the other two techniques. In conclusion, we propose that *Leishmania* and *T. brucei* present constitutive origins that are fired in every cell cycle, with further flexible sites of replication initiation being fired stochastically. Thus, the mechanisms that determine the latter sites of activation need to be determined, including whether the same strategies to complete DNA replication after potential replication–transcription conflicts are used in *T. brucei* and *Leishmania*. Indeed, the characteristics of constitutive origins, such as histone modifications that might recruit the initiator protein complexes or colocalization with centromeres, increasing the probability of these origins being fired, need to be addressed.

## Pre-Replication (Pre-RC) and Pre-Initiation (Pre-IC) Complexes

**Figure 2:**
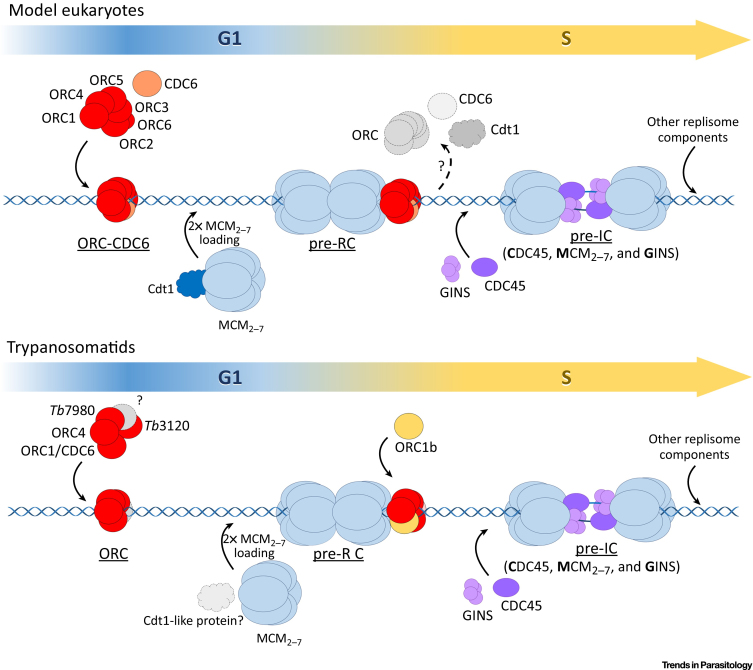
The Replication Initiation Machinery of Model Eukaryotes and Trypanosomatids. In model eukaryotes (top panel), from late mitosis to the end of G1 phase, the six-subunit ORC is recruited to all potential origins in the genome. Here, ORC interacts with CDC6. Next, the MCM_2–7_ helicase is recruited and loaded onto the origin by Cdt1. This forms the pre-RC, and renders the origins of replication ‘licensed’. At the onset of the S phase, CDC6 and Cdt1 are removed from the pre-RC, CDC45 and GINS are recruited to the origin, forming the pre-IC. Together, CDC45, the MCM_2–7_, and the GINS complex form the CMG complex, which is the active replicative helicase that unwinds the origin DNA, allowing the further assembly of the replicative fork components. In trypanosomatids (bottom panel), a divergent ORC-like complex is present, comprising ORC1/CDC6, ORC4, Tb3120, and Tb7980. It is not clear if other subunits remain to be identified. It is assumed that MCM_2–7_ is loaded, as in other eukaryotes, prior to the S phase, but how this is catalyzed is unclear, as no Cdt1 orthologue has been identified, and a clear orthologue of CDC6 remains undetermined. It remains possible that MCM is not loaded until the S phase (not shown). At the end of G1 and the start of the S phase, until late G2, an ORC1 orthologue, ORC1B, is expressed. CDC45 and the GINS complex are, most likely, then recruited to the origin, and the steps downstream are believed to take place in a similar way to model eukaryotes. ORC, origin recognition complex; CDC6, cell division cycle subunit 6; MCM, mini chromosome maintenance; Cdt1, CDC-dependent transcript 1; pre-RC, pre-replication complex; CDC45, cell division cycle subunit 45; GINS, ‘go-ichi-ni-san’ in reference to the proteins SId5, Psf1, Psf2, and Psf3; pre-IC, pre-initiation complex; CMG, complex composed of CDC45, MCM_2–7_, and GINS.

**Figure 3:**
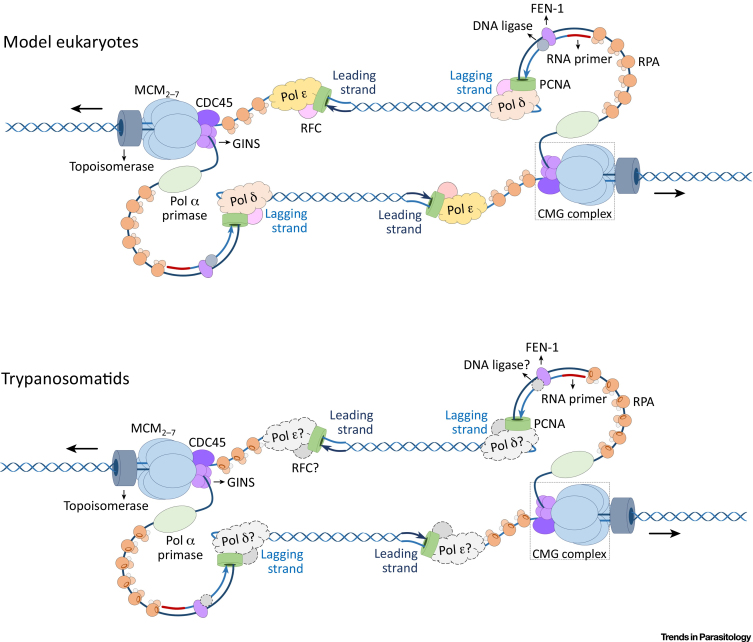
Replication Fork Progression in Trypanosomatids. All proteins belonging to the replication fork (topoisomerases, MCM_2–7_, CDC45, GINS, RPA, PCNA, and Pol α-primase) presented in color have been described in trypanosomatids. Genes encoding homologues of DNA polymerases δ and ε, RFC, and DNA ligase I (presented in gray, with a dashed border) are present in the sequenced genomes of trypanosomatid parasites, but their role in replication fork progression has not yet been characterized. MCM, mini chromosome maintenance; CDC45, cell division cycle subunit 45; GINS, ‘go-ichi-ni-san’ in reference to the proteins SId5, Psf1, Psf2, and Psf3; RPA, replication protein A; PCNA, proliferating cell nuclear antigen; RFC, replication factor C; FEN-1, flap endonuclease 1.

The establishment of a DNA replication origin occurs by the ordered recruitment of protein complexes (Figure 2), leading to the assembly of the replicative machinery. In model eukaryotes, DNA replication origins are demarcated by the binding of a conserved six-subunit initiator protein complex called the origin recognition complex (ORC), whose activity is modulated by the binding of another, related protein termed cell division cycle subunit 6 (CDC6). Five of the six ORC subunits (ORCs_1–5_) and CDC6 are AAA+ family ATPases and possess C-terminal winged helix (WH) DNA-binding domains. The ORC structure resembles an open two-layered ring with AAA+ ATPase subunits in one layer and WH domains in the other 16, 17. ORC6 is the only subunit that does not conform to the ORC AAA + –WH structure, and its location within the complex remains somewhat unclear, though it appears to be adjacent to ORCs2–3 18, 19. Binding of CDC6 between ORC1 and ORC2 appears to close the ring and allows ORC–CDC6 to recruit the replicative helicase called the mini-chromosome maintenance (MCM) complex, which is also composed of six AAA+ ATPase subunits (MCM_2–7_). Productive and stable interaction between ORC–CDC6 and MCM_2–7_ requires a further mediator, termed CDC-dependent transcript 1 (Cdt1), which can bind stably to MCM6 18, 19. Though structural studies show the MCM_2–7_ helicase is loaded as a double hexamer, and the precise mechanics of loading the two hexamers remain unclear, CDC6 also plays a central role in this reaction [20]. In total, ORC–CDC6–Cdt1–MCM_2–7_ (termed the prereplication complex, or pre-RC) assembles onto origins during the G1 phase. Activation of the pre-RC occurs in the S phase and involves interaction of each MCM_2–7_ hexamer with a single protein termed cell division cycle subunit 45 (CDC45) and a four-subunit complex called **GINS**. The resulting CDC45–MCM_2–7_–GINS (CMG) complex, also termed the pre-initiation complex (pre-IC), possesses helicase activity, and its formation is associated with MCM phosphorylation by the CDC7-Dbf4 kinase complex. Once formed, the CMG complex is able to recruit the other **replisome** components to melted DNA at the origin, a reaction that involves further factors, including MCM10 and a RecQ helicase (not shown in Figure 2, Figure 3).

Recent studies have begun to reveal pronounced divergence in the origin-binding initiator of trypanosomatids, when compared with model eukaryotes. In *T. brucei*, MCM_2–7_, CDC45, and GINS are conserved and essential (Box 2). Although functional analyses have not been extended beyond *T. brucei,* the conservation of the various components in *T. cruzi* and *Leishmania* suggest that the CMG machinery in trypanosomatids is very similar that in to model eukaryotes 21, 22. In contrast, trypanosomatid ORC has proved much more difficult to characterise. Initially, trypanosomatid genome searches identified only a single ORC-like factor (named ORC1/CDC6), homologous to ORC1 and CDC6 and capable of complementation of yeast CDC6 mutants [23]. Given the lack of identifiable orthologues of other ORC subunits, it was considered possible that ORC activity resides in a single factor, as is seen in members of the Archaea. Consistent with this hypothesis, no homologue of Cdt1 has yet been identified in any kinetoplastid. However, subsequent work revealed that *T. brucei,* and most likely all kinetoplastids, does in fact possess an ORC, albeit one that appears highly dissimilar to the conserved six-subunit ORC–CDC6 initiator seen in model eukaryotes (Figure 2). The first evidence of a diverged ORC came from the identification of a weakly conserved ORC1-like factor, which was named ORC1B and shown to interact with ORC1/CDC6 and MCM3 in *T. brucei* [22]. Later, three more ORC1/CDC6-interacting factors were identified in *T. brucei*: a highly divergent ORC4-like subunit, and Tb7980 and Tb3120, two factors with very limited primary sequence homology with ORC subunits [21]. Subsequently, RNAi revealed that loss of ORC1/CDC6, ORC1B, ORC4, or Tb3120 impedes DNA replication and leads to comparable growth and cell cycle defects [24]. Though loss of Tb7980 results in proliferation defects [21], clear evidence of a role in DNA replication has not yet been determined, but is likely. The strongest evidence for an ORC in *T. brucei* is found in the demonstration that ORC1/CDC6 and ORC4 are present in a high-molecular-weight complex (∼530 to 1011 kDa) that also seems to include MCM3, though whether all putative ORC and MCM subunits are also present is currently unknown [24]. Of note, localization studies suggest that ORC1B is unlikely to be a stable component of *T. brucei* ORC [24]. Nonetheless, ORC1/CDC6-binding sites have been mapped in the *T. brucei* genome and shown to colocalize with sites of replication initiation, clearly showing that at least one ORC component dictates constitutive origin function [10]. Around 60% of ORC1/CDC6 binding sites are found in subtelomeric chromosome regions containing **variant surface glycoprotein** (VSG) genes, where replication initiation has not been mapped. Whether this dense binding might relate to VSG expression control is currently unclear 25, 26.

**Figure I:**
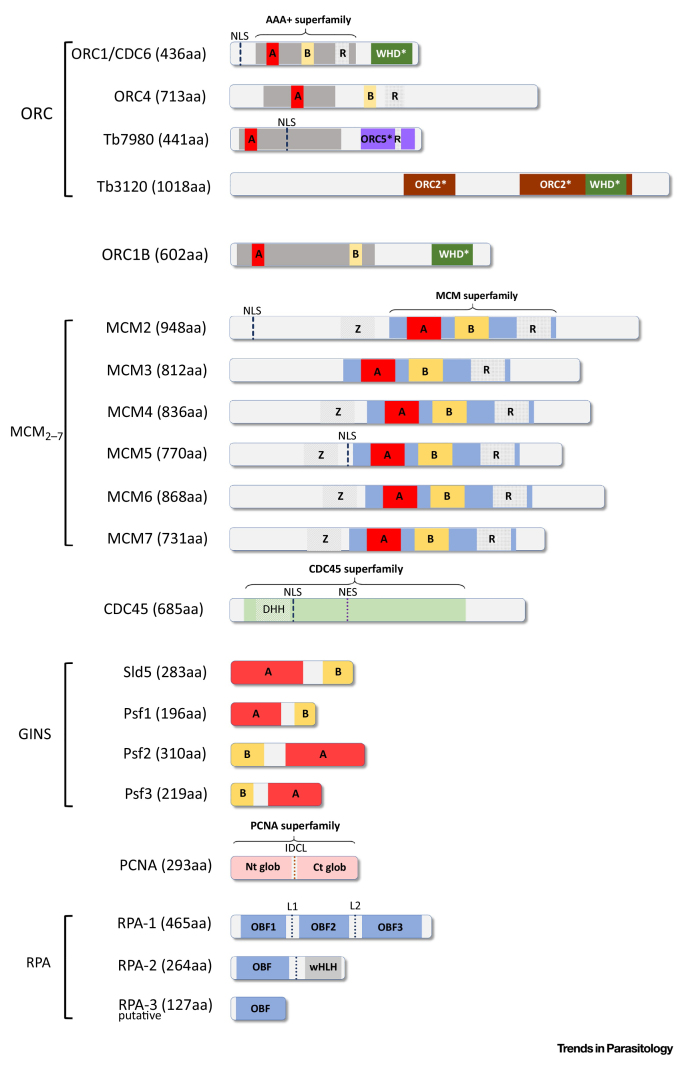
The Essential Domains and Motifs of the Main Proteins Involved in DNA Replication in Trypanosomatids. NLS, nuclear localization signal; NES, nuclear export signal; Z, zinc-finger motif; A and B, Walker A and Walker B motifs; R, arginine fingers motif; WHD*, putative winged helix domain; ORC5*, putative ORC5 domain with low significance; ORC2*, putative ORC2 domain with low significance; DHH, (Asp–His–His) motif; Nt and Ct glob, globular domains found at amino and carboxyl terminal regions of the protein; IDCL, inter-domain connecting loop; OBF, oligonucleotide/oligosaccharide-binding fold domain; wHLH, winged-helix-loop-helix domain; L, linkers between OBF domains.

Box 2 **Structural Analysis of the Proteins Involved in Replication**

Many homologues of replication proteins annotated in TriTrypDB possess structural differences compared to model eukaryotes.

(a) ORC-related proteins (ORC1/CDC6, ORC4, Tb7980, Tb3120, and ORC1b): reviewed in [21].
(b) CDC45: shares 20–5% identity with CDC45 from yeast and mammals. Despite the low identity, the conserved DHH domain is present [22]. Nuclear localization and export signals were also found in this counterpart [22].
(c) MCM_2–7_: all possess the conserved ATPase domain found in model eukaryotes, containing Walker A, Walker B, and arginine-finger motifs 22, 27. Moreover, a zinc-finger motif (important for double hexamer formation) was also found in the N-terminal region in five out of six MCMs, suggesting that this arrangement can be formed [22].
(d) GINS: organized by the arrangement of two conserved domains, A (rich in α helices) and B (rich in β strands). Sid5 and Psf1 contain a large A N-terminal domain and a small B C-terminal domain, while Psf2 and Psf3 possess these two domains in exactly the opposite order, which is the same domain organization described for other eukaryotes [22].
(e) PCNA: forms a homotrimeric ring complex and possesses conserved motifs, binding sites for DNA, and known interacting proteins, but contains an insertion of more than 30 residues in its C-terminal portion in all trypanosomatids 37, 99.
(f) RPA: composed of OB-fold domains similar to those in other eukaryotes but presents some important structural differences. The major subunit (RPA-1) lacks the first OB-fold domain (called 70N or DBD-F), which is important for protein–protein interactions 29, 44, 45. RPA-2 contains one OB-fold domain and one wHLH in its C-terminal region, and is very structurally related to the model RPA-2. Although a possible RPA-3 homologue that was predicted to contain an OB-fold domain was found at TriTrypDB, no reports about its structure and function are available [29].
(g) RFC and nuclear topoisomerases: although homologues of these proteins have been found in trypanosomatid genomes, no structural studies have been performed to date [8].
(h) CDC6 and Cdt1: homologs of these proteins were not found in trypanosomatid genomes.

A scheme showing the main domains and motifs for these proteins is represented in Figure I.

Alt-text: Box 2

ORC1B analysis suggests that the regulation of *T. brucei* (and therefore trypanosomatid) DNA replication may also be diverged. In contrast to ORC1/CDC6, ORC4, Tb3120, and Tb7980, which all localize to the *T. brucei* nucleus throughout the cell cycle, ORC1B localization and/or expression is highly cell-cycle-dependent and can only be detected in the nucleus of *T. brucei* cells in the S or G2 phase [24]. Thus, despite the sequence homology between ORC1B and ORC1 and/or CDC6, the expression and/or localization dynamics of this ORC-like factor do not resemble any eukaryotic ORC subunit thus far described (Figure 2). The active recruitment of ORC1B to the *T. brucei* nucleus during the S phase may indicate that the factor provides a positive regulatory role in DNA replication, which would be unprecedented [24]. How ORC1B might exert such an effect awaits experimental investigation, but its potential connection with the diverged kinetoplastid ORC is intriguing. The available data suggest that ORC1B is not a static member of the putative ORC-like complex and has drastically different expression dynamics to CDC6. However, is it possible that ORC1B provides modified CDC6-like functions? Binding of CDC6 converts ORC into a ring, allowing MCM_2–7_ recruitment in model eukaryotes. In this light, it is conceivable that the diverged kinetoplastid ORC needs ORC1B to ‘complete’ the complex, but this happens at the onset of the S phase and not prior to the S phase. If so, the replication initiation cascade might be radically different from model eukaryotes. For instance, ORC-MCM_2–7_ may interact and localize to origins in an inactive pre-RC that is only activated by binding of ORC1B (Figure 2). Alternatively, the pre-RC might only form at the outset of the S phase, when ORC1B allows MCM_2–7_ to interact; in other words, MCM_2–7_ is not recruited to origins until the S phase. Both possibilities may be consistent with the potential absence of Cdt1. However, they do not readily explain why ORC1/CDC6 is able to provide CDC6-like functions in being able to complement *Saccharomyces cerevisiae* CDC6 temperature-sensitive mutants [23]. Indeed, ORC1B has been suggested to lack ATPase activity, which perhaps renders it an unlikely candidate to mediate remodeling of the ORC [22]. Nonetheless, if any of these scenarios are correct, they raise questions that are presented in the outstanding questions box.

## Replication Fork Progression

After the establishment of a DNA replication origin and the assembly of the pre-IC complex, firing of replication occurs and replication is initiated with the bidirectional progression of replication forks from all origins. This process is conserved in the eukaryotic domain and, as trypanosomatids encode most of the predicted replication-fork proteins 22, 24, 27, 28, 29, we can update the description for replication fork progression in these organisms (Figure 3).

To open the double-stranded DNA and allow access to the DNA polymerases, trypanosomatids assemble the CMG complex [22]. RNA–DNA primers are generated by the **Pol α-primase complex**. Then, DNA polymerase delta (Pol δ) and epsilon (Pol ε) synthesize DNA on the lagging and leading strands, respectively. It should be noted, however, that, in trypanosomatids, the composition and activity of Pol δ, Pol ε, and Pol α-primase complexes remain to be fully characterized, though RNAi of a Pol α-primase in *T. brucei* results in S phase arrest [30]. To promote the processivity of the replicative polymerases, a DNA sliding clamp called proliferating cell nuclear antigen (PCNA) strengthens the interaction between the DNA template and the polymerases. In most eukaryotes, PCNA is recruited by replication factor C (RFC) 31, 32, 33, which has not been examined in trypanosomatids. In *Leishmania* spp. and *Trypanosoma cruzi*, PCNA is concentrated in the nuclear periphery during the S phase 34, 35, which is in agreement with localization of replicating DNA in the nuclear periphery 1, 36. On the other hand, *T. brucei* PCNA shows a dispersed pattern throughout the cell cycle [37], suggesting that DNA replication is organized differently within the nucleus of these trypanosomatids. PCNA and RFC form a moving platform for Pol δ and play a number of roles, such as increased processivity, binding to the primer terminus, and bridging to the other replication proteins 33, 38. Furthermore, RFC and PCNA act as a loading platform for the flap endonuclease-1 (FEN-1) and DNA ligase I, which process **Okazaki fragments** 33, 39. FEN-1 in *T. cruzi* was recently shown to participate in DNA replication and repair [40], but DNA ligase I has not yet been characterized in any trypanosomatid.

An essential protein for the progression of the DNA replication fork is replication protein A (RPA), which binds to ssDNA exposed at the replication fork to stabilize and protect this portion against nucleases. RPA helps in the recruitment of Pol α to replicate the region where RNA priming is required for initiation 41, 42 and reduces nucleotide misincorporation performed by Pol δ, Pol ε, and Pol α 41, 42, 43. In trypanosomatids, the best-characterized component of RPA is RPA-1, which lacks a 70N domain that is responsible for the interaction with repair proteins and protein kinases in model organisms 29, 44, 45, 46, 47. In *T. cruzi*, RPA-1 and RPA-2 participate in DNA replication, while in *Leishmania amazonensis* RPA-1 seems to participate in double-strand break (DSB) repair, since it colocalizes with Rad51, a protein that catalyses homologous recombination 29, 44, 45.

Topoisomerases, which participate in resolving the over- or under-winding of DNA, constitute another group of proteins essential during replication fork progression. In trypanosomatids, topoisomerase type I is associated with the nucleolus and nuclear **chromatin** 33, 48, while topoisomerase type II is essential for nuclear and kinetoplast DNA replication. Topoisomerase II proteins from *T. cruzi* and *Leishmania donovani* have ATP-dependent and ATP-independent **decatenating activities** 49, 50, 51. In *L. donovani*, topoisomerase II appears in the nucleus and the **kinetoplast**, while in *T. cruzi* it is found only in the nucleus 33, 49, 50, 51. In most trypanosomatids, topoisomerase II shares high sequence identity and functions almost exclusively as a mitochondrial enzyme. However, two nuclear topoisomerase II enzymes (TbTOP2α and TbTOP2β) were described in *T. brucei*. They share similarity with nuclear topoisomerases from other eukaryotes, but TbTOP2α encodes an ATP-dependent topoisomerase, whereas the role played by TbTOP2β remains unclear [51].

Under normal conditions, the replication fork of trypanosomatids, like all eukaryotes, continues synthesizing DNA until it reaches the chromosome **telomeres**. Due to the inability of the Pol α-primase complex to initiate the last round of Okazaki fragment synthesis efficiently [52], telomeres exhibit a protrusion called a 3′ overhang, which acts as a substrate for the enzyme **telomerase** to elongate the telomeres [53]. Of note, trypanosomatids differ in the protein components that bind single- or double-stranded telomeric regions, while retaining the same telomere repeat nucleotide structure, (TTAGGG)_n,_ found in most eukaryotes 33, 54, 55.

The overall conservation in the replisome complexes in trypanosomatids, compared with model eukaryotes, is evident when we compare the speed of the DNA replication fork. Recent studies were able to calculate the replication speed in the different trypanosomatids using DNA combing: 1.84 kb/min for *T. brucei* strain Lister 427 [13], 3.7 kb/min for *T. brucei* strain TREU927 [12], 2.48 kb/min for *L. mexicana* [13], 2.45 kb/min for *L. major* [13], and 2.37 kb/min for *L. donovani* [13]. These rates range from 1.8 to 3.7 kb/min, which is broadly similar to the replication rates found in yeast (1.6 kb/min) [56], mouse embryonic fibroblasts (1.16 kb/min) [13], and in various human cell lines (∼1–2 kb/min) 57, 58. The small potential fork rate increase, relative to other eukaryotes, and potential differences between trypanosomatids, may be related to chromatin structure and function, reflecting multigenic transcription and the observation that trypanosomatid histones are divergent from those found in yeast and vertebrates and may undergo specific modifications 8, 59, 60.

## Mechanisms That Prevent Replication

While DNA replication is fundamental for cell proliferation, blockage of additional replication during and after the S phase is imperative for genomic stability. Hence, the firing of origins within replicated regions should be avoided during the cell cycle. The compartmentalization of origin licensing at the G1 phase and origin firing at S phase is a perfect strategy to inhibit re-replication, because the S phase cyclin-dependent kinase (CDK) can fire origins at the same time as controlling pre-RC components to limit re-initiation at origins that have already fired. This control includes the modulation of pre-RC component expression and subcellular localization, in addition to their ability to interact with DNA. In yeast, CDC6 and Cdt1 are phosphorylated by CDK during the transition to G1/S phase, and these modifications can trigger protein degradation 61, 62, 63. Non-DNA-bound MCM_2–7_ is exported to the cytoplasm after the S phase [64]. Additionally, phosphorylation of ORC2 and ORC6 by CDK 65, 66, as well as the interaction of CDK with CDC6, inhibits the recruitment of Cdt1/MCM onto origins [67]. Finally, the inhibition of all MCM subunits by **sumoylation** prevents helicase activation, which negatively regulates replication [68]. In metazoans, ORC1 and CDC6 undergo proteolysis after phosphorylation by CDK, and CDC6 is exported to the cytoplasm after CDK phosphorylation [69]. Phosphorylated MCM_2–7_ is not exported to the cytoplasm but has a reduced activity for binding origins [70]. Since Cdt1 has an essential role in recruiting MCM_2–7_ onto origins, metazoan cells rely on geminin, a Cdt1 inhibitor, to restrict replication to the S phase [71].

Re-replication events in the eukaryotic genome may lead to chromosome and gene copy number variation, which is a prevalent phenomenon in trypanosomatids, especially in the genus *Leishmania* [9]. However, other than ORC1B, no modulation of pre-RC component expression or subcellular localization has been described for any trypanosomatid. ORC1/CDC6 is present in the nuclear space in *L. major*, and remains bound to DNA throughout the cell cycle in *T. brucei* and *T. cruzi* 23, 72. In *T. brucei*, MCM_2–7_ also remains in the nucleus throughout the cell cycle [22]. At this stage, due to the divergence of the pre-RC in trypanosomatids, it is not simple to infer how these organisms control DNA re-replication. Post-translational modifications, such as phosphorylation by CDKs, could act by avoiding helicase loading onto DNA after the S phase, but no protein kinase that controls DNA replication has been described. Moreover, as noted above, the unusual expression or localization of ORC1B, and the potential absence of Cdt1, has not been explored mechanistically in the context of replication control. Nonetheless, it has been shown that CDC45, a component of the pre-IC which activates MCM helicase activity together with GINS complex, is exported from the nucleus after the S phase in *T. brucei* [22]. Therefore, this may be a mechanism to prevent DNA re-replication, but how it occurs is unknown. DNA re-replication in other eukaryotes can lead to the generation of DNA DSBs and activation of the DNA damage checkpoint [73]. Considering that the potential mechanisms of gene amplification in *Leishmania* require factors that act in DSB repair [9], we can speculate on the existence of a possibly lax control to avoid the DNA re-replication and the pervasive structural variation exhibited by the genomes of *Leishmania* spp. It is possible that the same may apply to *T. cruzi*, where chromosome ploidy variation has been observed [74], though underlying mechanisms have not been explored. Chromosome ploidy changes in *T. brucei* appear limited to the VSG-rich subtelomeres, perhaps indicating a more rigid control of re-replication than in the other trypanosomatids [75].

In addition to replication prevention post-S phase, the DNA replication process is usually turned off in the infective forms of trypanosomatids. For instance, in *T. cruzi* trypomastigotes (infective form), ORC1/CDC6 is located in the nuclear space, but is not able to bind DNA. Moreover, MCM7 is not expressed in these parasite forms [27]. These data suggest that pre-RC components might be key factors in establishing replication arrest during the *T. cruzi* life cycle. Furthermore, *T. cruzi* probably uses different strategies to prevent DNA replication at the non-S phases of the replicative forms or in the infective ones, since ORC1/CDC6 is bound throughout the cell cycle in replicative forms but not bound to DNA in infective ones. The efficacy and details of these strategies, as well as their relation with the infection efficiency of *T. cruzi* and other trypanosomatids, requires further investigation.

## DNA Replication Stress

**Figure 4:**
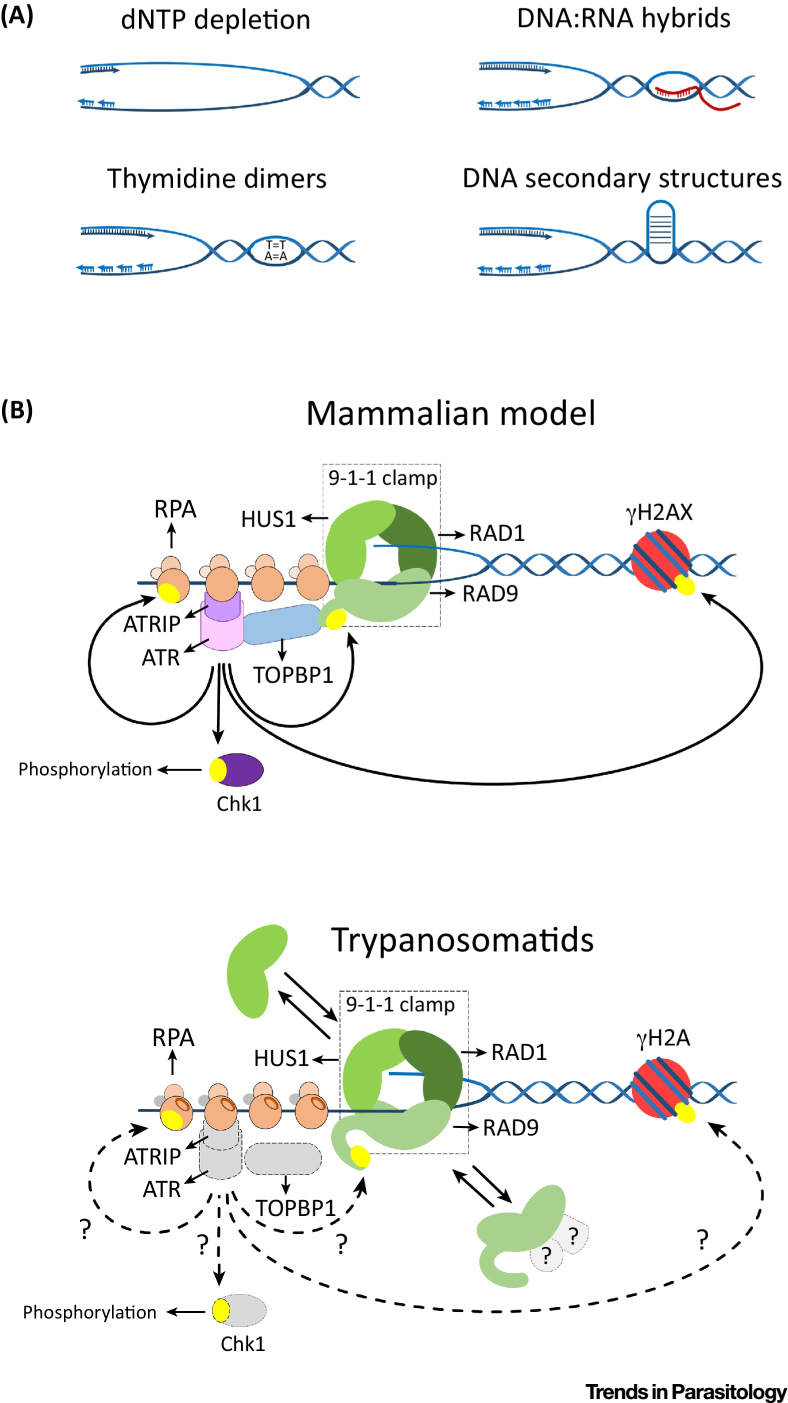
The Replication Stress Response in Mammals and Trypanosomatids. (A) Obstacles that can slow or halt DNA replication leading to replication stress. These include dNTP depletion, DNA lesions (such as thymidine dimers), DNA secondary structures, and DNA:RNA hybrids. In all of these circumstances, the replicative helicase uncouples from the DNA polymerases, causing the accumulation of ssDNA and triggering replication stress-response. (B) The mammalian (top panel) and trypanosomatid (bottom panel) models for the replication stress response. Top panel. In this model, ATR kinase is recruited to RPA-coated ssDNA through its binding partner ATRIP. As an independent event, the 9-1-1 clamp is loaded at the ssDNA–dsDNA junction. TopBP1 is also recruited to RPA–ssDNA at the ssDNA–dsDNA junctions, interacts with the C-terminal tail of Rad9, and stabilizes the ATR kinase localization at the site of stress. ATR phosphorylates downstream factors, including H2A(X) and Chk1, which mediates cell cycle arrest and controls origin firing. Bottom panel. Most of the data for this pathway were observed in *Leishmania.* These parasites express a functional 9-1-1 homolog; the Rad9 subunit is found in alternative complexes, and Hus1 also exists as a monomer, suggesting a functional flexibility and compartmentalization of the trypanosomatid 9-1-1 clamp. Homologs of key elements of this pathway have not yet been characterized, such as the ATR–ATRIP complex, TOPBP1, or Chk1 (dashed gray molecules). dNTP, deoxynucleotide; ssDNA, single-stranded DNA; ATR, ataxia telangiectasia Rad3-related; RPA, replication protein A; ATRIP, ATR-interacting protein; Rad9, radiation sensitive subtype 9; Rad1, radiation sensitive subtype 1; Hus1, checkpoint protein HUS1; 9-1-1, complex composed of Rad9, Rad1, and Hus1; dsDNA, double-stranded DNA; TopBP1, DNA topoisomerase 2-binding protein 1; H2AX, histone variant; Chk1, checkpoint kinase 1.

Replication stress is determined by events in which the DNA replication fork is slowed or halted, such as a decreased nucleotide pool, certain types of DNA lesions, RNA/DNA hybrids, or DNA secondary structures (Figure 4A). For some of these obstacles, there are translesion DNA Pols that help to overcome the DNA lesions [30]. However, in most cases the replicative helicase uncouples from the DNA Pol and continues to unwind the DNA duplex, inducing the accumulation of long stretches of single-stranded DNA (ssDNA) that are protected from degradation by association with RPA [76]. RPA-coated ssDNA mediates the recruitment of the ataxia telangiectasia Rad3-related (ATR) protein kinase and its binding partner ATR-interacting protein (ATRIP) [77]. The ATR–ATRIP complex helps to stabilize and restart the collapsed fork and promotes the phosphorylation of substrates that include histone H2A(X) and checkpoint kinase 1 (Chk1) (Figure 4B) 78, 79. During these reactions, the ssDNA/dsDNA junction in these structures mediates the loading of a protein complex composed of Rad9, Rad1, and Hus1 (also called 9-1-1 clamp) [80]. The heterotrimeric 9-1-1 complex facilitates the recruitment of DNA topoisomerase 2-binding protein 1 (TopBP1), which interacts with the C-terminal domain of Rad9 and activates ATR–ATRIP (Figure 4B) 81, 82, 83. This set of reactions ultimately leads to cell cycle arrest and ensures the conclusion of DNA synthesis.

Trypanosomatids dwell in inhospitable environments in which replication stress may arise and cause a threat to their genome’s stability. *Leishmania* spp. replicate under conditions of high oxidative stress within host macrophages 54, 84, *T. brucei* proliferates in the host bloodstream [85], and *T. cruzi* multiplication is inhibited for long periods in chronic infections [86]. The genome architecture and transcription mechanism of trypanosomatids may favor replication stress: because virtually all genes are cotranscribed from multigene transcription units that may include hundreds of genes 2, 87, the long-distance and near constitutive movement of RNA Pol II across the genome must increase the severity of replication–transcription conflicts [88].

The trypanosomatid genome encodes many homologous proteins of the replication stress response, and their functional characterization, which has only begun, has already revealed remarkable features. As mentioned before, trypanosomatid RPA-1 lacks the 70N domain (Box 2), which in other eukaryotes is required for RPA binding to the 9-1-1 clamp and ATR activation [89]. In *Leishmania*, RPA-1 associates with chromatin in response to replication stress and colocalizes with Hus1 and Rad9 [90]. It is possible that the interplay between RPA, 9-1-1, and ATR is mediated by another still unknown protein in this parasite, which could not only supply the absent 70N domain but also serve as a distinct point of regulation for the replication stress response.

Homologs of all the 9-1-1 subunits have been identified in trypanosomatids. In *Leishmania*, all three subunits associate with chromatin in response to replication stress, and Rad9- or Hus1-deficiency impacts replication stress and DSB responses [90]. Primary sequence and structural predictions indicate that the degree of conservation is not the same for the three subunits, suggesting the existence of a distinct selective pressure that drives the evolution of these subunits in *Leishmania*. Rad9 is the most divergent subunit with an expanded C-terminal domain [91], which harbors key phosphorylation sites required for the genotoxic stress response in other eukaryotes 92, 93. Such structural divergence of Rad9 suggests that the functional regulation of 9-1-1 might also present relevant peculiarities. Mammals and yeast have evolved paralogs and isoforms of 9-1-1 subunits, which possibly allow them to engage in compartmentalized functions 94, 95. In *Leishmania*, the apparent absence of 9-1-1 paralogs has possibly been compensated for by the formation of alternative complexes [90]. Two sets of evidence corroborate this idea. First, Rad9 exists in a complex distinct from 9-1-1, and Hus1 is found in a monomeric form. Second, the phenotypes observed in response to replication stress and DSB formation in Rad9-deficient cells are distinct from those associated with Hus1 deficiency. Hence, functional flexibility of the 9-1-1 complex in *Leishmania* is probably enabled by the association of the subunits in different complexes within the cell.

Currently, it is unknown whether ATR is required for the replication stress response of trypanosomatids, but kinase activity is necessary for the replication stress response in *Leishmania*. For instance, phosphorylation of H2A is triggered by replication stress in a Rad9- and Hus1-dependent manner [90], but the specific kinase activity linked to this process has not been identified [96]. Another common response to replication stress in eukaryotes is activation of dormant origins 97, 98. A recent study found evidence that replication stress can trigger activation of at least one putative dormant origin in *T. brucei* [12], but this has not been expanded to a genome-wide scale or to other trypanosomatids. Furthermore, no *T. brucei* proteins or pathways that act in dormant origin activation have been identified and, as we have noted above, we cannot rule out origin-independent initiation of replication after stress, perhaps in *Leishmania* or *T. cruzi* particularly.

## Concluding Remarks

There are many differences emerging in the structural composition of the protein complexes involved in DNA maintenance between trypanosomatids and eukaryotic models, though in most cases the advantages and disadvantages of the presence or absence of particular domains and/or motifs in specific proteins remains unknown (see Outstanding Questions). What was previously thought to be a single-component ORC complex (ORC1/CDC6), is now likely a multimeric complex. In addition, further studies are necessary to reveal the complete composition of the pre-RC, as well as the action of ORC1B. There is insufficient evidence to infer the mechanism used by trypanosomatids to prevent re-replication, although clues point to CDC45 as a possible inhibitor of replication outside the S phase [22]. On the other hand, there is evidence pointing to pre-RC components as key factors in blocking DNA replication in infective forms of *T. cruzi* [27]. In addition, we have only started unveiling the replication stress response in these parasites, and the establishment of a unique response to this phenomenon may have shaped some of the peculiar genome phenomena observed in these parasites, including the remarkable expression of VSG genes in *T. brucei* and the extraordinary genome plasticity found in *Leishmania* species. Further understanding of the pre-RC and 9-1-1 components, as well as identification of pathways implicated in the response to replication stress, may provide a basis for the design of more effective chemotherapy arsenals against the devastating diseases caused by trypanosomatids.

Outstanding Questions Are there functional homologs for CDC6 and Cdt1 in trypanosomatids?

Could the ORC complex recruit MCM_2–7_ and bind to DNA without ORC1B?

Is there a need for kinase-mediated regulation of the trypanosomatid pre-RC if ORC1B provides a positive, activating function?

If ORC remains bound to the origins after replisome recruitment, how does the replisome bypass it?

Why is the DNA replication rate in trypanosomatids slightly higher compared with model eukaryotes?

What is the mechanism used by these protozoans to avoid DNA re-replication during and after the S phase?

Are there other proteins involved in the recruitment of the 9-1-1 clamp and activation of ATR in response to replication stress?

What are the advantages or disadvantages of the divergent structures of some replication machinery proteins?

What mechanisms are used by trypanosomatids to resolve the predicted severe conflicts between transcription and replication that arise due to multigenic transcription?

Does the execution and/or control of genome replication lead to genome plasticity in trypanosomatids?

Do trypanosomatids use widespread origin-independent replication initiation to complete synthesis of their genomes?

## Glossary

Chromatin: complexes of macromolecules on the nuclear genome of eukaryotic cells.
Constitutive origins: DNA replication origins that are activated in all cells of a population.
Decatenating activities: the process of separating topologically-linked circular chromosomes.
Dormant origins: DNA replication origins that are not fired during a normal cell cycle but are activated in the presence of DNA damage/replication stress.
Flexible origins: DNA replication origins whose use varies from cell to cell in an apparently stochastic manner.
GINS: an acronym from the first letters of the Japanese numbers 5-1-2-3 (go-ichi-ni-san) in a reference to the proteins SId5, Psf1, Psf2, and Psf3.
Kinetoplast: a unique mitochondrial DNA structure organized in a giant network of interlocked rings (also called kDNA), found exclusively in flagellated protists belonging to the group Kinetoplastea.
Okazaki fragments: short, newly synthesized DNA fragments (with an RNA primer on the 5′ end) generated on the lagging strand during DNA replication.
Pol α-primase complex: DNA polymerase alpha is an enzyme complex from eukaryotes composed of four subunits: POLA1 (catalytic subunit), POLA2 (regulatory subunit), PRIM1 (small primase subunit), and PRIM2 (large primase subunit).
Replisome: complex machinery composed of several proteins, including MCM_2–7_, CDC45, GINS, RFC, PCNA, RPA, topoisomerases, pol α-primase, pol δ, pol ε, FEN-1, and DNA ligase. Its main function is to carry out replication of DNA.
Sumoylation: a post-translational protein modification by addition of SUMOs (small ubiquitin-like modifiers).
Telomerase: a reverse transcriptase enzyme responsible for elongating telomeres by addition of a species-dependent telomere repeat sequence to the 3′ overhang.
Telomeres: a region at the end of eukaryotic chromosomes, composed of repetitive sequences and associated proteins, whose main function is to protect the chromosome from the action of exonucleases and from fusion with other chromosomes.
Variant surface glycoprotein genes: genes which encode proteins that pack the cell surface of *T. brucei* and allow this parasite to evade the host’s immune system by antigenic variation.
